# Opening the black box: interpretable machine learning for predictor finding of metabolic syndrome

**DOI:** 10.1186/s12902-022-01121-4

**Published:** 2022-08-26

**Authors:** Yan Zhang, Xiaoxu Zhang, Jaina Razbek, Deyang Li, Wenjun Xia, Liangliang Bao, Hongkai Mao, Mayisha Daken, Mingqin Cao

**Affiliations:** Department of Epidemiology and Health Statistics, College of Public Health, Xinjiang Medical University, Urumqi, Xinjiang China

**Keywords:** Metabolic syndrome, Data mining, Machine learning, Model interpretability, Risk prediction model

## Abstract

**Objective:**

The internal workings ofmachine learning algorithms are complex and considered as low-interpretation "black box" models, making it difficult for domain experts to understand and trust these complex models. The study uses metabolic syndrome (MetS) as the entry point to analyze and evaluate the application value of model interpretability methods in dealing with difficult interpretation of predictive models.

**Methods:**

The study collects data from a chain of health examination institution in Urumqi from 2017 ~ 2019, and performs 39,134 remaining data after preprocessing such as deletion and filling. RFE is used for feature selection to reduce redundancy; MetS risk prediction models (logistic, random forest, XGBoost) are built based on a feature subset, and accuracy, sensitivity, specificity, Youden index, and AUROC value are used to evaluate the model classification performance; post-hoc model-agnostic interpretation methods (variable importance, LIME) are used to interpret the results of the predictive model.

**Results:**

Eighteen physical examination indicators are screened out by RFE, which can effectively solve the problem of physical examination data redundancy. Random forest and XGBoost models have higher accuracy, sensitivity, specificity, Youden index, and AUROC values compared with logistic regression. XGBoost models have higher sensitivity, Youden index, and AUROC values compared with random forest. The study uses variable importance, LIME and PDP for global and local interpretation of the optimal MetS risk prediction model (XGBoost), and different interpretation methods have different insights into the interpretation of model results, which are more flexible in model selection and can visualize the process and reasons for the model to make decisions. The interpretable risk prediction model in this study can help to identify risk factors associated with MetS, and the results showed that in addition to the traditional risk factors such as overweight and obesity, hyperglycemia, hypertension, and dyslipidemia, MetS was also associated with other factors, including age, creatinine, uric acid, and alkaline phosphatase.

**Conclusion:**

The model interpretability methods are applied to the black box model, which can not only realize the flexibility of model application, but also make up for the uninterpretable defects of the model. Model interpretability methods can be used as a novel means of identifying variables that are more likely to be good predictors.

**Supplementary Information:**

The online version contains supplementary material available at 10.1186/s12902-022-01121-4.

## Introduction

Data mining is recognized as a fast and effective method to obtain information and create knowledge from complex big data, and has shown good performance and broad application prospects in health examination big data research. However, the internal workings of machine learning classification algorithms are complex and considered as low-interpretation "black-box" models, and the process of making decisions by the models cannot be visualized and transparently demonstrated in most cases, which makes it difficult for the application personnel to understand and trust these complex models [1]. In addition, most models developed by data scientists primarily use prediction accuracy as a performance evaluation metric and rarely interpret their predictions in a meaningful way [2]. Especially for complex black box models such as random forests and neural networks, although the accuracy is high, the interpretability is low, and it is difficult to explain the model results in a reasonable and intuitive way if the model prediction results are used to replace the decision making by doctors. It is thus clear that the problem of model uninterpretability limits the practical application of machine learning in the clinical setting, and therefore, it is imperative to address the problem of model interpretability.

Different classifications of interpretation methods can be made based on different criteria, grouping them according to when they are applied: before, during, and after building a machine learning model [3]. Pre-model interpretability techniques usually occur before model is established, are model-independent, and apply only to the data itself, since it is also important to explore and fully understand the data before modeling, and meaningful intuitive features and sparsity (a small number of features) help to achieve some of the properties of data interpretability. The interpretability in the model involves the machine learning model, which has inherent interpretability. Post-model interpretability refers to improving interpretability after the model has been built (post hoc). In addition, another important distinction is model-specific and model-agnostic. Model-specific interpretation methods are restricted to specific models, e.g., the interpretation of weights in a linear model is model-specific, and by definition, the interpretation of an inherently interpretable model is always model-specific. The model-agnostic approach can be applied to any machine learning model, applied after the model is trained, relying on the inputs and outputs of the analytic pair of elements. It is characterized by the possibility of interpreting the model without sacrificing its predictive power [4].

Feature selection (FS), as an important data pre-processing technique, enables interpretability before modeling. FS constructs a subset of the original feature set and does not change the physical meaning of the features [5]. In related studies [6, 7], it is shown that FS methods reduce the dimensionality of data by removing redundant and irrelevant data features, which can reduce the complexity of models and increase their comprehensibility to some extent. In addition, a popular approach in current research is to interpret the model after building it, that is, a post hoc model-agnostic interpretation method, which is an interpretation method independent of the training model. Even if the prediction results are obtained through a "black box" model, the use of post hoc-assisted attribution interpretation and visualization tools enables explanatory studies of the model [8–10], which can help the application personnel understand the process and reason of the model's decision-making.

Metabolic syndrome (MetS) is a group of disease syndromes with metabolic abnormalities characterized by centripetal obesity, hypertension, hyperglycemia and dyslipidemia [11]. The prevalence of MetS has shown an increasing trend due to rapid economic growth, aging population, sedentary lifestyle, and obesity. Globally, the prevalence of MS is about 20–25% [12]. In China, the standardized prevalence of MetS is about 24.2% in the adult population [13] and about 34.0% in the middle-aged and elderly population [14]. MetS leads to an increased risk of diabetes, cardiovascular disease, cancer, and even death [15, 16] and has become an increasingly serious public health problem and clinical challenge [17]. Therefore, appropriate prevention and control strategies must be adopted to reduce the incidence of MetS. Health checkups are the first stage of disease prevention, and data mining of physical examination information can help identify people at high risk of MetS at an early stage, thus moving the gateway to disease prevention and control. The construction of MetS risk prediction models based on physical examination data is important for the prevention and control of MetS.

The study uses data mining methods to construct MetS risk prediction models based on physical examination data, with MetS as the entry point. Feature selection method is used to select key factors associated with MetS from numerous physical examination indicators; focuses on post hoc interpretability to increase the practical application value of MetS risk prediction models. The study can accurately predict and identify high-risk individuals and provide information reference for the prevention and control of MetS, and at the same time, it can provide methodological reference for the feasibility of applying feature selection combined with model interpretability methods in medical examination data mining.

## Methods

### Data source

The data are obtained from a chain of health screening institutions in Urumqi, Xinjiang Uygur Autonomous Region, China, for people who underwent routine health screening from 2017 ~ 2019. The study was approved by the Ethics Committee of the First Affiliated Hospital of Xinjiang Medical University, all methods were carried out in accordance with relevant guidelines and regulations. The physical examination information included basic demographic information, questionnaire surveys, routine physical examination, and laboratory physiological and biochemical index tests.

Questionnaire survey: A self-designed questionnaire is used to conduct a face-to-face survey by uniformly trained investigators, which includes gender, age, ethnicity, smoking status (never smoked; smoking means those who still smoked in the past 30 days at the time of the survey; quit means no longer smoked in the past 30 days at the time of the survey), alcohol consumption (never drank; Occasional drinking refers to drinking < 1 time/week in the past 1 year; regular drinking refers to drinking ≥ 1 time/week in the past 1 year; quit drinking refers to no longer drinking in the past 30 days), previous disease history (hypertension, diabetes, etc.) and family history (hypertension, diabetes).

Physical examination: height, weight, waist circumference (WC), heart rate and blood pressure are measured using a uniform instrument, the instrument is calibrated before measurement, and the measurement parameters of height, weight and WC are accurate to 0.1 kg or 0.1 cm. Blood pressure is measured using an electronic automatic blood pressure measuring instrument, and the subjects avoid strenuous exercise and caffeinated beverages for 30 min before measurement, and rest for at least 5 min before the first measurement, with an interval of 1 to 2 min between each measurement. Body mass index (BMI) = weight (kg)/height (m^2^).

>Laboratory tests: 10 mL of fasting venous blood is drawn from the study subjects in the early morning, and the physiological and biochemical indexes such as blood routine, fasting plasma glucose (FPG), blood lipids, liver function and kidney function are measured by automatic biochemical analyzer.

Diagnosis of MetS: with reference to the diagnostic criteria for MetS recommended in the Chinese Guidelines for the Prevention and Treatment of Dyslipidemia in Adults (2016 Revised Edition) [18], MetS can be diagnosed if at least three of the following items are met.

- ① Central obesity or abdominal obesity: WC ≥ 90 cm in men and ≥ 85 cm in women.
- ② Hyperglycemia: FPG ≥ 6.10 mmol/L or those who have been diagnosed and treated for diabetes mellitus.
- ③ Hypertension: systolic blood pressure (SBP) ≥130 mmHg or diastolic blood pressure (DBP) ≥85 mmHg or those who have been diagnosed and treated for hypertension.
- ④ Fasting triglycerides (TG) ≥ 1.7 mmol/L.
- ⑤ Fasting high density lipoprotein cholesterol (HDL-C) < 1.04mmol/L.

### Data pre-processing

The original physical examination data contains rich physical examination information, but also contains various " corrupted data", for example, data entry errors resulting in abnormal values and missing values, which can increase the complexity and difficulty of statistical analysis, therefore, data cleaning and sorting are performed before data analysis. A total of 44,547 medical examiners' information is collected for the study, and the medical examination data are checked for outliers, and 21 cases of outlier data (e.g., age = 178 years, height = 2.56 m, etc.) are removed. The data of 5392 cases with missing diagnostic variables of MetS are deleted, and finally 39,134 physical examination data are left. Other missing data in the physical examination data are filled using multivariate imputation chained equations (MICE). MICE belongs to the multiple interpolation technique, which is a popular method for handling missing data with flexibility and robustness characteristics [19].

### Feature selection

FS is a common and effective feature reduction method when selecting a suitable low-dimensional subset from an initial high-dimensional dataset [20–22]. Recursive feature elimination (RFE) belongs to wrapper method in the feature selection method, which is a method that relies on the learning algorithm and uses the results of the learning algorithm as evaluation criteria to select a subset of features [23]. RFE uses a machine learning model to perform multiple rounds of training, eliminating a number of features corresponding to the weight coefficients at the end of each round, and then performing the next round based on the new set of features. The performance of the RFE algorithm depends on which classifier is used for the iteration.

The RFE steps are as follows:

- ① Initializing the feature set $F$.
- ② Select the classifier *C*.
- ③ Calculate the weight of each feature ${f}_{i}$ in $F$ (the criterion is the accuracy of the classifier prediction).
- ④ Remove the minimum weight feature ${f}_{i}$ and update $F$.
- ⑤ Repeat steps ③ and ④ until only one feature remains in $F$.
- ⑥ Feature importance ranking.

### Data mining prediction models

Three MetS risk prediction models, logistic regression (LR), random forest (RF), and extreme gradient boosting (XGBoost), are constructed using whether the study subjects had MetS as the target variable and each influencing factor as the input variable to compare and evaluate the robustness of predictive classification models.

#### Logistic regression

LR is one of the classical regression modeling methods with advantages in interpreting model results and computational costs [24], and is widely used in medicine and epidemiology. The MetS target variable is assumed to be a binary variable taking values of no disease(X = 0) and disease(X = 1). P(y = 1|X) denotes the probability of an individual developing disease when the exposure factor is X, the ratio of the probability of disease (*P*) to the probability of no disease (1-*P*) is the odds ratio (OR) and logit(P) is the natural logarithm of OR.

1 $$\mathrm{logit}(P)=ln\left(\frac{P}{1-P}\right)$$

The LR model:

2 $$\mathrm{logit}\left(P\right)=\mathrm{\alpha }+\sum_{j=1}^{k}{\beta }_{j}{x}_{j}$$

#### Random forest

RF is an integrated learning algorithm based on statistical learning theory proposed by Breiman [25] in 2001, which is essentially a combinatorial classifier containing multiple decision trees. Random forest combines Bootstrap resampling technique and decision trees to construct a collection of tree classifiers containing multiple basic classifiers, and the category with more decision votes $H(x)$ is used as the category to which the final sample belongs, using a simple majority voting method.

#### Extreme Gradient Boosting

XGBoost is a boosted tree model, which is based on multiple decision trees, using gradient boosting as a framework and stages in a way to combine multiple weak classifiers, using a minimization loss function to create strong classifiers. The objective function during training consists of two parts, the first part is the gradient boosting algorithm loss and the second part is the regularization term, the loss function is defined as:

3 $$L\left(\phi \right)=\sum_{i}l\left({\widehat{y}}_{i},{y}_{i}\right)+\sum_{k}\Omega ({f}_{k})$$

$l$ is the loss for a single sample, which is assumed to be a convex function to measure the difference between the prediction ${\widehat{y}}_{i}$ and the target ${y}_{i}$.

The complexity of the model is defined using the regularization term:

4 $$\Omega \left(f\right)=\gamma T+\frac{1}{2}\lambda {\Vert w\Vert }^{2}$$

$\gamma$ and $\lambda$ are manually set parameters, $w$ is the vector formed by the values of the leaf nodes of the decision tree, and $T$ is the number of leaf nodes.

### Post hoc model-agnostic interpretation methods

Post hoc model-agnostic interpretation methods are divided into global interpretability and local interpretability. A crucial aspect of dividing the interpretability methods is based on the scale of interpretation, where local interpretability providesan explanation only for a specific instance, and global interpretability explains the whole model [26]. Global interpretability helps to understand the modeling relationship and distribution of the predicted target based on the input variables, and local interpretability helps to understand the model prediction of a single instance [26, 27]. The two methods used in combination can mutually explain the decision results of the model. The study conducted the global interpretation of the model through variable importance and partial dependence plot (PDP), and local interpretable model-agnostic explanations (LIME) for local interpretation.

#### Variable importance

Variable importance measures the contribution of each input variable by the increase in the prediction error of the model after displacing the variable [28], and a feature is considered important if displacing it increases the error rate (reduces performance) [29]. The basic principle of variable importance is to calculate the predicted value after perturbation by perturbing a feature ${x}_{j}$and comparing the new feature value with the original feature value; the larger the difference shows that the variable is more important.

The calculation method:

- ① Input the trained model, the feature matrix *X*, the target vector *Y*, and the error function $L(Y,\widehat{Y})$.
- ② Calculate the original prediction error.
- ③ For each feature $(j=1, 2, \cdots p)$, generate the perturbed feature matrix ${X}_{permj}$ by perturbing the *j* feature.
- ④ Calculate the new error ${e}_{perm}\left(\widehat{f}\right)=L(Y,\widehat{f}\left({X}_{permj}\right))$.
- ⑤ Calculating the importance parameter ${FI}_{j}=\frac{{e}_{perm}\left(\widehat{f}\right)}{{e}_{orig}\left(\widehat{f}\right)}$, or ${FI}_{j}={e}_{perm}\left(\widehat{f}\right)-{e}_{orig}(\widehat{f})$.
- ⑥ Arrange each ${FI}_{j}$ by size.

#### Partial Dependence Plot

PDP shows the marginal impact of features on the prediction results of a machine learning model and helps to visualize the relationship between variables and prediction results [30, 31]. PDP relies on the model itself and requires training the model first (e.g., training the XGBoost model) and then interpreting a feature based on the model in relation to the target variables based on the model. The partial correlation function of the regression is defined as:

5 $${\widehat{f}}_{{x}_{S}}\left({x}_{S}\right)={E}_{{x}_{C}}\left[\widehat{f}\left({x}_{S},{x}_{C}\right)\right]=\int \widehat{f}\left({x}_{S},{x}_{C}\right)d{\mathbb{P}}\left({x}_{C}\right)$$

The set ${x}_{S}$ is the dependent variable for which the PDP is to be drawn, and ${x}_{S}$ usually contains one or two features; ${x}_{C}$ is the rest of the dependent variables used in the machine learning model $\widehat{f}$. The dependent variables in ${x}_{C}$ are marginalized so that only the relationship between the dependent variable and the variables in ${x}_{S}$ is shown.

Assuming that the relationship between the target variable and feature ${X}_{1}$ is to be studied, then the PDP is about the predicted value of the model as a function of feature ${X}_{1}$. The XGBoost model ($XGB\_model$) is first fitted, and then the i-th feature of the k-th sample in the training set is denoted by ${X}_{i}^{k}$. The bias function is estimated by a Monte Carlo method, that is, the average of the *N* instances of the training data is calculated as follows:

6
$$\int \left({X}_{i}\right)=\frac{1}{n}\sum_{k=1}^{n}XGB\_model\left({X}_{1},{X}_{2}^{k},{X}_{3}^{k},\cdots ,{X}_{n}^{k}\right)$$

#### Locally interpretable model-agnostic explanations

LIME is a post hoc local explanation method that uses locally interpretable models (linear models, decision trees, etc.) to explain the individual predictions of any black box machine learning model (in the vicinity of the prediction to be explained instances) [32]. The LIME approach proceeds by adding a slight perturbation to the input sample, observing the change in the output of the black box model, determining the degree of influence of different features on the prediction results by the degree of change, and then assigning weights based on the distance between the perturbed data points and the original data to train an interpretable model based on the perturbed sample. LIME generates an interpretation of instance $x$ according to Eq. 7:

7
$$explanation\left(x\right)=\begin{array}{c}argmin\\ g\epsilon G\end{array}\mathcal{L}\left(f,g,{\pi }_{x}\right)+\Omega \left(g\right)$$

where G is a class of interpretable (linear) models, an ensemble of simple models; $f$is the model to be interpreted; $\mathcal{L}$ is the loss function that minimizes the function; ${\pi }_{x}$ is the proximity measure between instances z and $x$ (kernel defines locality); and $\Omega \left(g\right)$ is an optional regularization term to control (limit) the model complexity.

### Statistical processing

Excel 2019 software is used to establish a data warehouse, to summarize and organize the physical examination data, and R software (version 3.6.0, http://www.r-project.org) was applied for statistical analysis. The MICE method was first used to fill in the missing data, and then the RFE method in the feature selection method was used for variable screening. The MetS risk prediction model was constructed based on LR, RF and XGBoost models, and the performance of the model was evaluated based on accuracy, sensitivity, specificity, Youden index [ 33] and area under the receiver operating characteristic curve (AUROC), with all values ranging from 0 to 1. The closer to 1, the better the model prediction performance. The definitions and formulas for accuracy, sensitivity, specificity, and Youden index are provided in Supplementary file S 3. The AUROC values and 95%CIs of the models were calculated and compared using MedCalc statistical software (version 15.6.1, https://www.medcalc.org), where the 95% CIs of the AUROC values were calculated using the binomial exact confidence interval method and the differences in the AUROC values were compared using the DeLong method [ 34]. Finally, the post hoc interpretability of the model is studied based on variable importance, PDP and LIME.

The study randomly divides 39,134 cases of research subjects into training set (70%) and test set (30%) according to the ratio of 7:3. The prediction model is constructed by the training set, and the model effect evaluation is carried out by the test set. Among them, 27,394 cases in the training set, 4080 cases (14.9%) are diagnosed with MetS, and 11,740 cases in the test set, 1693 cases (14.4%) were diagnosed with MetS.

## Results

### Feature selection

**Fig. 1:**
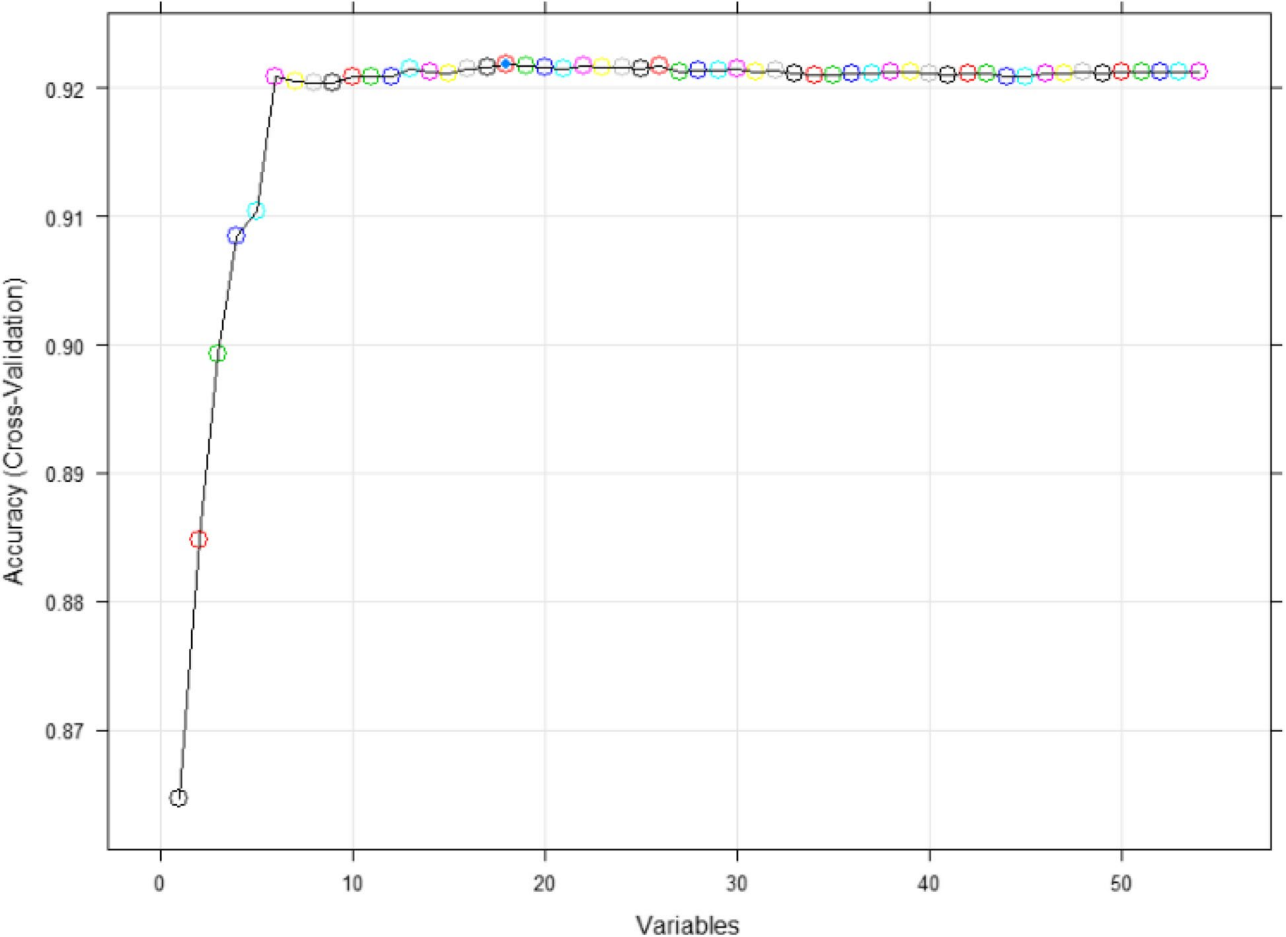
RFE cross-validation result curve.A point in the graph represents a variable, which is a different variable

The cross-validation result curve of the accuracy of RFE screening variables is shown in Fig. 1, which shows that the highest accuracy and better feature selection effect was achieved when the number of variables was 18, and the variables screened were: WC, HDL-C, TG, FPG, previous diabetes, SBP, gender, previous fatty liver, DBP, age, previous hypertension, uric acid, glutamyl transpeptidase, total cholesterol (TC), alkaline phosphatase, creatinine, erythrocyte distribution width coefficient of variation, eosinophil percentage.

### Construction of MetS risk prediction model

With the subset of features screened by RFE as input variables, and whether to have MetS as the target variable (Y: 1 = yes, 0 = no), three MetS risk prediction models were constructed by logistic, random forest, and XGBoost, respectively.

#### Based on feature selection dataset

**Fig. 2:**
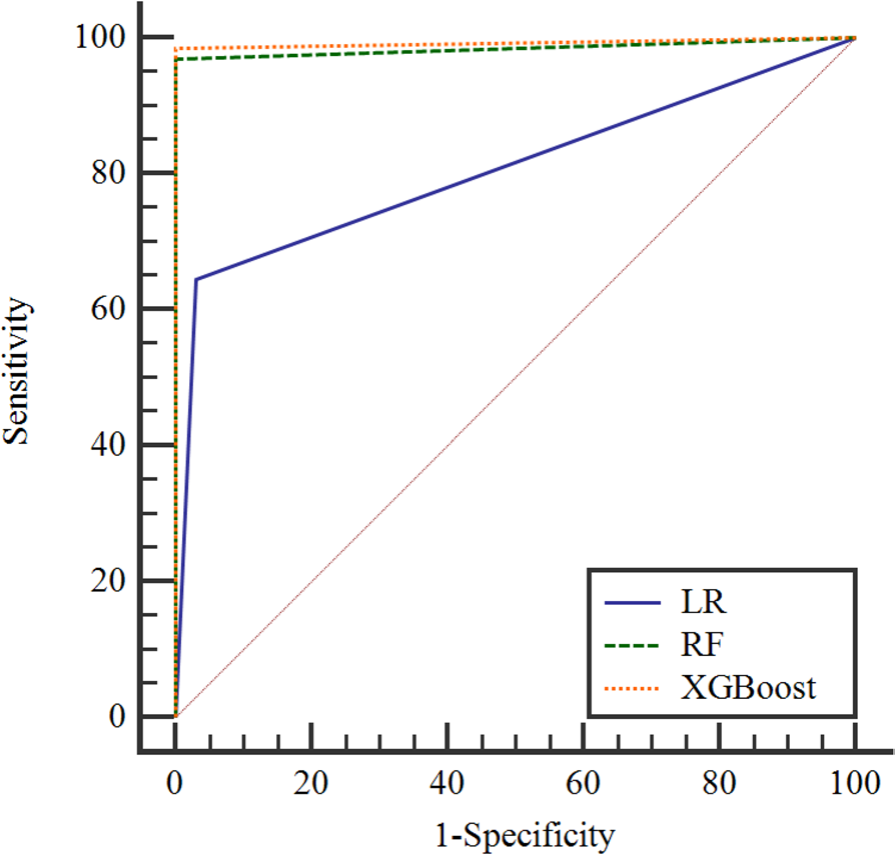
ROC curve of MetS risk prediction model in the test set

According to Table 1, the performance evaluation results of constructing MetS risk prediction models based on RFE feature subset showed that RF and XGBoost models had higher accuracy, sensitivity, specificity, Youden index, and AUROC values compared with logistic regression, and XGBoost models have higher sensitivity, Youden index, and AUROC values compare with RF. The ROC curve plots of LR, RF and XGBoost models based on the subset of RFE features show that the ROC curve of XGBoost model is closest to the upper left corner of the coordinate axis and has a higher AUROC value, as shown in Fig. 2.

**Table 1:** Performance evaluation of MetS risk prediction in the test set

Classification model	Accuracy(%)	Sensitivity(%)	Specificity(%)	Youden index	AUROC (95%*CI*)
LR	92.3	64.5	97.0	0.615	0.807(0.800 ~ 0.815)^a^
RF	99.5	96.9	100	0.969	0.984(0.982 ~ 0.987)^b^
XGBoost	99.7	98.5	99.9	0.984	**0.992**(0.990 ~ 0.993)

^a^indicates AUROC values of the XGBoost model compared with LR, Z = 30.986,*P*< 0.001

^b^indicates AUROC values of the XGBoost model compared with RF, Z = 3.920,*P*< 0.001

#### Research on the interpretability of risk prediction models

Since the XGBoost model is a better classification model, the study uses the variable importance, PDP and LIME to study the interpretability of the XGBoost model.

#### Importance of variables

**Fig. 3:**
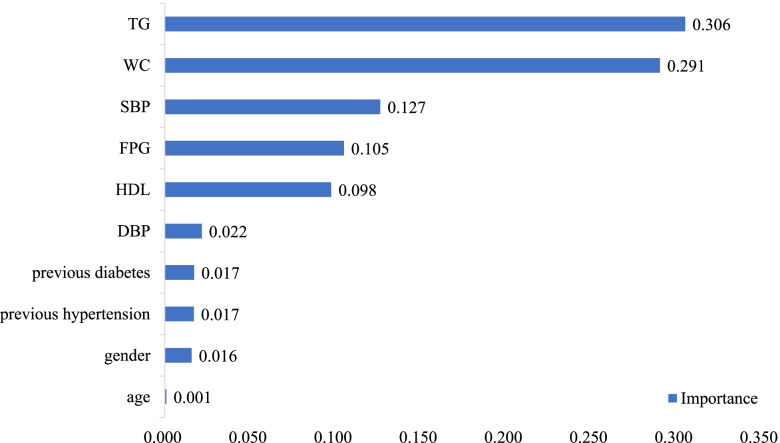
Variable importance of XGBoost model based on training set showing the top 10 variables

Figure 3 shows the 10 most important variables in the XGBoost model construction process, in descending order of importance: TG, WC, SBP, FPG, HDL-C, DBP, previous diabetes, previous hypertension, gender, and age.

#### LIME

**Fig. 4:**
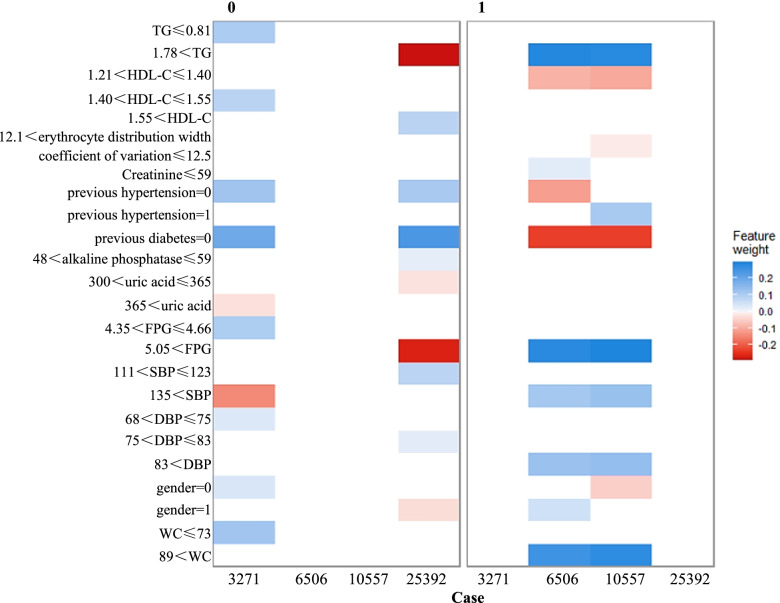
Visualized heat map of the variable combination of four medical examiners (training set) based on LIME. The direction of feature action is shown by color, blue (feature weight > 0) means the feature supports the outcome variable, red (feature weight < 0) means the feature opposes the outcome variable; the color shade refers to the degree of influence of the feature on the outcome variable, and the dark color indicates that the feature has a large influence on the metabolic syndrome

**Fig. 5:**
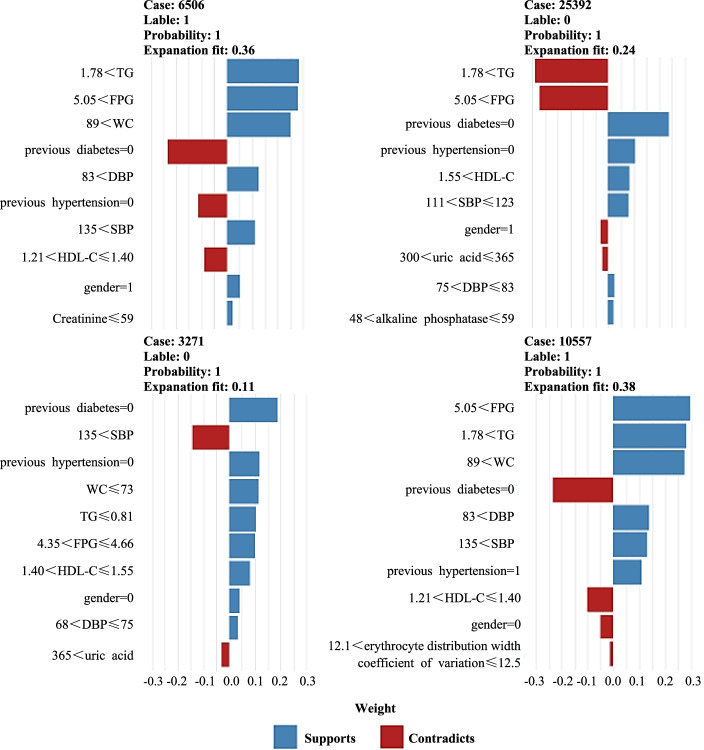
Interpretation of individual prediction (training set) based on LIME diagram. The length of the bars is proportional to the strength of the characteristic effect

Two subjects with MetS and two subjects without MetS are randomly selected from the training set subjects, and the specific data of the four subjects are shown in Table 2. The visualized heat map of the combination of variables for the four subjects based on the LIME method is shown in Fig. 4, and the interpretation of the predicted values for the four subjects individually is shown in Fig. 5, which shows the 10 most important variables associated with the occurrence of MetS and the 10 most important variables without MetS, respectively, as well as the direction and intensity of the effect of each influencing factor on the outcome, for example, triglycerides > 1.78 mmol/L is shown in red in the left graph as an opposing factor without MetS and in blue in the right graph as a supporting factor for MetS, so triglycerides > 1.78 mmol/L is a risk factor for the occurrence of MetS.

**Table 2:** Specific data for 4 subjects in the training set

**Variables**	**Physical examiner number**			
	3271	6506	25,392	10,557
Gender (0 = female, 1 = male)	0	1	1	0
Age (years)	26	61	45	59
eosinophil percentage	8.3	3.3	2.6	1.6
erythrocyte distribution width coefficient of variation	12.4	14.4	12.9	12.2
creatinine (μmoI/L)	79	52	54	72
uric acid (μmoI/L)	386	260	304	373
glutamyl transpeptidase (U/L)	32	44	16	48
alkaline phosphatase (U/L)	48	115	56	69
previous fatty liver (0 = no, 1 = yes)	0	1	1	1
previous hypertension (0 = no, 1 = yes)	0	0	0	1
previous diabetes (0 = no, 1 = yes)	0	0	0	0
WC (cm)	72	91	84	90
SBP (mmHg)	139	154	122	169
DBP (mmHg)	71	85	83	109
FPG (mmol/L)	4.4	5.13	5.18	6.38
TC (mmol/L)	3.76	5.16	5.19	6.22
TG (mmol/L)	0.77	2.49	1.79	2.98
HDL-C (mmol/L)	1.54	1.22	1.67	1.33
MetS (0 = no, 1 = yes)	0	1	0	1

Combining the LIME results, we can obtain: TG, HDL-C, erythrocyte distribution width coefficient of variation, previous hypertension, previous diabetes, alkaline phosphatase, FPG, SBP, DBP, gender, WC, and uric acid are associated with MetS, with TG ≤ 0.81 mmol/L, HDL-C > 1.21 mmol/L, 12.1 < erythrocyte distribution width coefficient of variation ≤ 13.0, no previous hypertension, no previous diabetes, 48 U/L < alkaline phosphatase ≤ 59 U/L, 4.35 mmol/L < FPG ≤ 4.66 mmol/L, 111 mmHg < SBP ≤ 123 mmHg, 68 mmHg < DBP ≤ 83 mmHg, female, and WC < 73 cm are protective factors for the development of MetS; TG > 1.78 mmol/L, creatinine ≤ 59 μmoI/L, previous hypertension, uric acid > 300 μmol/L, FPG > 5.05 mmol/L, SBP > 135 mmHg, DBP > 83 mmHg, male, and WC > 89 cm are risk factors for the development of MetS.

#### Partial Dependence Plot

**Fig. 6:**
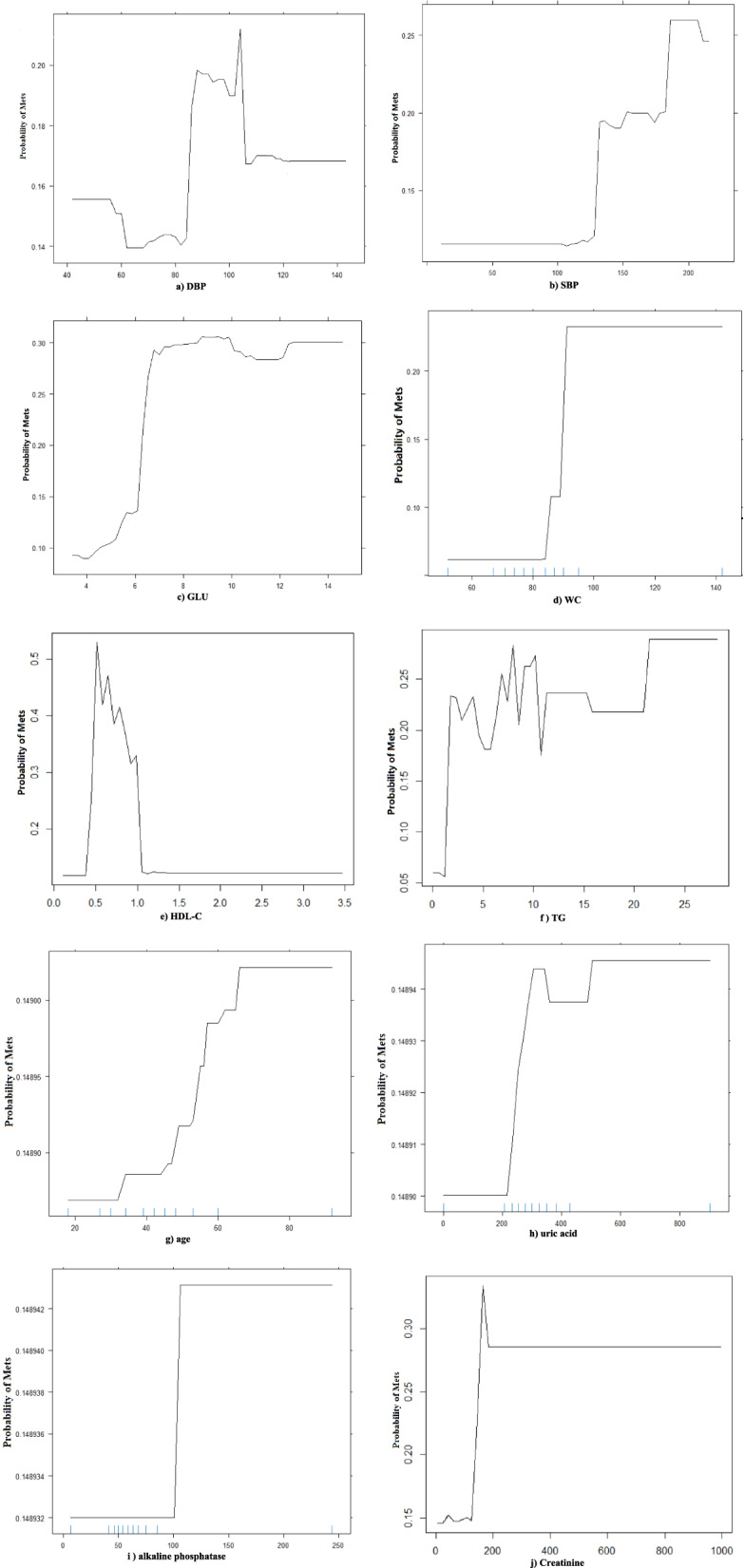
PDP diagram of important variables in the XGBoost model (training set)

From variables importance and LIME, it can be obtained that the important variables associated with MetS include: TG, WC, SBP, FPG, HDL-C, DBP, age, creatinine, alkaline phosphatase, previous diabetes, previous hypertension, and gender, and the relationship between the continuous variables and the predicted probability of MetS was visualized using PDP plots, as shown in Fig. 6. From the figure, it can be concluded that a nonlinear relationship was observed between each variable and the probability of MetS occurrence.

- The probability of MetS in subjects with DBP between 68 and 83 mmHg is lower than that of DBP < 60 mmHg and DBP > 83 mmHg, and the probability of MetS at DBP around 83 mmHg is significantly higher than that of MetS at DBP < 60 mmHg.
- The probability of MetS is low when SBP < 110 mmHg of the subject, and the probability of MetS begins to increase when the SBP is around 110 mmHg, and increases significantly when the SBP > 135 mmHg.
- The probability of MetS in the subject increases with increasing FPG, and at FPG > 7 mmol/L, the probability of MetS stabilizes and shows small fluctuations with increasing FPG.
- The probability of MetS in the subjects increases with increasing WC and tends to stabilize at WC > 90 cm.
- The probability of MetS in the subject begins to decrease at HDL-C around 0.5 mmol/L, decreasing to the lowest probability and stabilizing at 1.21 mmol/L.
- The probability of MetS is low at TG < 1.7 mmol/L in subjects, and the probability of MetS increased significantly at about 1.7 mmol/L. After TG > 1.7 mmol/L, the probability of MetS showed small fluctuations as TG increased.
- The probability of MetS is low at age < 35 years, increases with age at age ≥ 35 years, and stabilizes at about 70 years.
- The probability of MetS increases significantly at around 200 umol/L uric acid and stabilizes at around 500 umol/L.
- The probability of MetS is low for alkaline phosphatase < around 100 U/L and significantly higher and stabilized at around 100 U/L.
- The risk of MetS is higher at creatinine ≤ 59 μmoI/L than at creatinine 59–130 μmoI/L, and the probability of MetS is significantly higher and stabilized at around 130 μmoI/L.

## Discussion

### Application value of risk prediction model

In recent years, with the development of computer science and technology, various types of risk prediction models have been widely used in various fields of medicine. Risk prediction models use statistical models to estimate the risk of developing future outcomes for individuals based on one or more underlying characteristics [35]. Healthcare interventions or lifestyle changes are targeted to those at increased risk of developing the disease. These models can also be to screen individuals to identify those who are at an increased risk of having an undiagnosed condition, for which diagnosis management and treatment can be initiated and ultimately improve patient outcomes [36]. Šoštarič A et al. [37] used logistic regression models to construct a prediction model for MetS based on lifestyle, simple anthropometric indicators and blood parameters for identifying young individuals with increased risk of MetS, and the model had good interpretability. Kanegae H [ 38] used 18,258 patients' health examination data from 2005–2016 to build prediction models based on machine learning methods (XGBoost, ensemble learning) and traditional statistical methods (logistic regression), and according to the test dataset model results showed that the AUROC values of XGBoost, ensemble learning and logistic regression models were 0.877, 0.881 and 0.859, respectively, and the prediction performance of machine learning models was better than that of traditional statistical models; Chang W [39] proposed a prediction method for prognostic outcomes based on physical examination indicators in hypertensive patients, using four classification algorithms: support vector machine, C4.5 decision tree, random forest and XGBoost to predict patients' prognosis, and among the four classifiers XGBoost had the best prediction performance with accuracy, F1 and AUROC values of 94.36%, 0.875 and 0.927. The machine learning models showed superior predictive performance in the related studies, but the transparency and interpretability of the models were low.

The study found that compared with logistic regression model, Random Forest and XGBoost model both have better classification prediction performance. Logistic regression is a classical approach in statistics and the most commonly used model for disease risk prediction, which requires many important assumptions to be satisfied in its application (e.g., independence of observations and no multicollinearity between variables). In contrast, machine learning algorithms make fewer assumptions about the underlying data, which results in algorithms that are usually more accurate for prediction and classification [40]. In addition, machine learning relies on computers to learn the complex nonlinear interactions between variables by minimizing the error between predictions and observations [41, 42], and therefore, machine learning algorithms have shown superior performance in most studies. Among the three MetS risk prediction models, the XGBoost model has the best predictive performance, which is similar to the results of Congxin Dai et al. [43]. Some scholars have shown that the high flexibility that XGBoost allows for fine-tuning may make its performance slightly better than random forest [44]. XGBoost uses parallelization and distributed computing to ensure efficient computing time and resources. It is an optimization model that combines a linear model with a Boosting tree model, using not only the first derivative of the loss function but also the second derivative of the loss function to reduce the possibility of overfitting, adjusting for errors generated by existing models and improving their effectiveness [45]. However, the functional relationship between the input and output of XGBoost model is difficult to understand, especially in medical applications, where the "black box" property of the model may make the model unpredictable and risky or make biased decisions.

### Application value of model interpretability methods

The research focuses on the interpretation of models after they are built, that is, post-hoc model-agnostic interpretation methods, which are used to interpret complex machine learning prediction models and can help application personnel understand the process and rationale for the decisions made by the models. Variable importance and PDP provide global explanations. Variable importance quantifies the relationship between the independent and dependent variables in the model and visually shows the relative strength of the independent variables' influence on the model. PDP is graphical representations of predictive functions that help visualize the relationship between variables and predicted outcomes, and can show whether the relationship between objectives and features is linear, monotonic, or more complex. For example, when applied to a linear regression model, PDP always shows a linear relationship. However, LIME is a local interpretation of the model, which can be interpreted for each individual's prediction results, suggesting specific cut-off values for disease risk factors, which is more early warning for individual disease prevention than logistic regression. But the disadvantage of its application is the instability of interpretation [46]. The variable importance, PDP, and LIME methods have the characteristics of freedom and flexibility in the choice of models compared with the nomograms that are often used currently. The nomogram is a transparent and interpretable analysis based on a specific model, which builds on logistic regression analysis and transforms complex regression equations into visual graphs that intuitively show the contribution of predictor variables to the results, making the results of the predictive model more readable [47]. Therefore, the black box model combined with the model interpretability method can not only realize the flexibility of model application, but also make up for the uninterpretable defects of the model, which will help to accurately find high-risk individuals with MetS from the physical examination data.

Different interpretability techniques can reveal different insights into the behavior of the model, where global interpretation can enable clinicians to understand the entire conditional distribution modeled by the trained response function. On the contrary, local interpretation can promote a partial understanding of the conditional distribution of a particular instance. Various interpretability techniques may interpret the behavior of machine learning models differently. The advantage of global interpretability technology is that it can be extended to the entire population, suggesting the general trend of influencing factors on the outcome, while local interpretability technology focuses on interpretation at the instance level and can facilitate insight into the predicted outcomes for a particular research object. According to the needs of the application, these two methods can be equally effective, and both are effective methods to assist clinicians in the medical decision-making process.

### Factors influencing the risk of developing MetS

According to the diagnostic criteria for MetS proposed by the World Health Organization (WHO), the Adult Treatment National Cholesterol Education Program Group (ATP III), the European Group for Insulin Resistance Research (EGIR) and the International Diabetes Federation (IDF), the included components are WC, BMI, TG, HDL-C, FPG and blood pressure, which are risk factors for MetS. In addition, MetS has been reported to be associated with other possible risk factors in related studies. The interpretable risk prediction model in this study can help to identify risk factors associated with MetS, and the results showed that in addition to the traditional risk factors such as overweight and obesity, hyperglycemia, hypertension, and dyslipidemia, MetS was also associated with other factors, including age, creatinine, uric acid, and alkaline phosphatase.

Studies have found that age is positively correlated with the risk of MetS. The probability of MetS increased with age when the age was ≥ 35 years, and the probability of MetS stabilized at about the age of 70 years, which was roughly similar to the results of related studies. Wang S [48] showed that age was a significant predictor of MetS in the working population, with older individuals having a higher risk of developing MetS. In a survey of the prevalence of MetS in the United States from 2003 to 2012, a comparison of the prevalence of MetS based on three age groups, 20–39, 40–59, and ≥ 60 years old, showed that the prevalence of MetS increased with age [49]. So far, the mechanism of the association between serum creatinine and MetS is unclear. The results of a cross-sectional study of 1,017 consecutive morbidly obese patients showed a negative association between serum creatinine and T2DM when serum creatinine levels were below 69 and 72 μmol/l in women and men, respectively [50]. More recently, Kengo Moriyama [51] found that the ratio of serum uric acid to creatinine was associated with a higher risk of MetS. Our study showed segmental changes in the association between serum creatinine and MetS. The risk of MetS was higher in physical examiners with creatinine ≤ 59 μmoI/L than in those with creatinine of 59 to 130 μmoI/L, and the risk of MetS increased significantly at about 130 μmoI/L and then stabilized. This result is complementary to the current study.

This study showed that the probability of MetS was higher at alkaline phosphatase levels greater than about 100 U/L, suggesting that high levels of alkaline phosphatase are a risk factor for MetS. An association between serum alkaline phosphatase activity and MetS has been reported by researchers, but this association has not received a uniform answer. In a community-based cross-sectional survey of the association between osteocalcin and MetS in Korean men and postmenopausal women, the association between alkaline phosphatase activity and MetS was found to be statistically insignificant after adjustment for age, BMI, and osteocalcin [52]. Furthermore, in another nationally representative cross-sectional study, high levels of alkaline phosphatase were associated with a high prevalence of MetS after adjusting for potential confounding variables [53]. Several mechanisms could explain the significant relationship between serum alkaline phosphatase activity and MetS, and although the pathophysiology of the MetS is not fully understood, insulin resistance and subclinical low-grade inflammation play a key role in the development of the MetS [53]. The results of this study showed that the probability of developing MetS was significantly higher in physical examiners with uric acid greater than 200 umol/L. Uric acid is the final enzymatic product of purine metabolism in the body, and related studies suggest that hyperuricemia, as an independent risk factor for atherosclerosis and coronary heart disease, is closely associated with many risk factors for MetS (e.g., obesity, abnormal lipid metabolism, hypertension, etc.) [54].

Our study also has some limitations. First, the study is based on a cross-sectional study, Machine learning models combined with interpretable methods can help identify factors associated with MetS that may be associated with prognostication and risk stratification in healthy populations, but cannot be justified. Second, the study used the LIME method to interpret the individual prediction results, but the interpretation of the results for two study individuals with very close values differed significantly, and the method still has the shortcoming of instability at present.

## Conclusion

Based on health examination data, the study takes MetS as the entry point and uses data mining classification models combined with model interpretability methods to build high classification performance and easy-to-understand MetS risk prediction models. The interpretability methods can be used as a novel means of identifying variables that are more likely to be good predictors. These predictors can be evaluated as features in other models developed with more appropriate datasets. In addition, it can also provide a methodological reference for the feasibility of applying the model interpretability method in health examination data mining.

## Supplementary Information

**Additional file 1:Supplementary file S1.**The original dataset for this study.

**Additional file 2: Supplementary file S2.** The dataset processed by MICE method for this study.

**Additional file 3: Supplementary file S3.** Definitions and formulas for accuracy, sensitivity, specificity, and Youden index.

## Data Availability

The dataset for this study is provided in supplementary file S1 and S2.

## Abbreviations

FS: Feature selection
MetS: Metabolic syndrome
WC: Waist circumference
BMI: Body mass index
FPG: Fasting plasma glucose
SBP: Systolic blood pressure
DBP: Diastolic blood pressure
TG: Triglycerides
HDL-C: High density lipoprotein cholesterol
MICE: Multivariate imputation chained equations
RFE: Recursive feature elimination
LR: Logistic regression
RF: Random forest
XGBoost: Extreme gradient boosting
PDP: Partial dependence plot
LIME: Local interpretable model-agnostic explanations
AUC: Area under the receiver operating characteristic curve
