# Supplementary material for: Opening the black box: interpretable machine learning for predictor finding of metabolic syndrome
Source: BMC Endocr Disord. 2022 Aug 26;22:214. doi: 10.1186/s12902-022-01121-4 (PMC9419421; doi:10.1186/s12902-022-01121-4)
Supplement: Supplementary file 3 — Additional file 3: Supplementary file S3. Definitions and formulas for accuracy, sensitivity, specificity, and Youden index. [file 12902_2022_1121_MOESM3_ESM.docx]

**Definitions and formulas for accuracy, sensitivity, specificity, and Youden index.**

| **Confusion matrix for two classification problems** | | |
| --- | --- | --- |
| Actual classification | Predicted classification | |
|  | ＋ | — |
| ＋ | True Positive (TP) | False Negative (FN) |
| — | False Positive (FP) | True Negative (TN) |
| TP is the number of positive samples correctly predicted by the classification model; FN is the number of positive samples incorrectly predicted as negative classes by the classification model;  FP is the number of negative samples incorrectly predicted as positive class by the classification model;  TN is the number of negative samples correctly predicted by the classification model. | | |

(1) Accuracy: The number of all correctly classified samples divided by the total number of samples.

$$\text{Accuracy}=\frac{TP+TN}{N}\times100\%$$

(2) Sensitivity: The proportion of correct predictions among all samples with positive class predictions.

$$\text{Sensitivity}=\frac{TP}{TP+FN}\times100\%$$

(3) Specificity: The proportion of correct predictions among all samples with negative predictions.

$$\text{Specificity}=\frac{TN}{TN+FP}\times100\%$$

(4) Youden index: If sensitivity and specificity are equally important, the Youden index can be applied, which is the sum of sensitivity and specificity minus 1. The larger the value, the higher the truthfulness of the predicted classification.

$$Youden index=\text{sensitivity}+\text{specificity}-1$$
